# Concept and Implementation of Measurement Systems for Stationary and Remote Testing of Sensors for Electrical and Non-Electrical Quantities

**DOI:** 10.3390/s23041928

**Published:** 2023-02-08

**Authors:** Leszek Furmankiewicz, Mirosław Kozioł, Ryszard Rybski, Robert Szulim

**Affiliations:** Institute of Metrology, Electronics and Computer Science, University of Zielona Góra, 65-417 Zielona Góra, Poland

**Keywords:** distance learning, laboratory stand, current sensors, displacement sensors

## Abstract

In intelligent transportation, various types of sensors are used both in traffic control systems as well as in the control, safety, and entertainment systems of the vehicles themselves. In the process of educating future designers and developers of such systems, it is necessary to familiarize them with the operation and parameters of sensors. The recent years of the COVID-19 pandemic have disturbed this process due to the need to conduct classes remotely. This article presents the general concept of a laboratory stand for testing sensors of electrical and non-electrical quantities, which can be used both in stationary and remote learning. Additionally, the practical implementation of two laboratory stands for testing current and linear displacement sensors was also presented. Both stands have been tested in the remote access mode. The tests showed some shortcomings in the management software but also confirmed the correctness of the adopted concept of their implementation.

## 1. Introduction

Due to the growing number of all types of vehicles, a significant increase in interest in the development of intelligent transport has been observed for many years. The concept of intelligent transport is often understood very broadly and applies not only to vehicles but also to the accompanying infrastructure. In practice, intelligent transport is a series of systems that should ensure the safety, mobility, efficiency, small impact on the environment, and customer satisfaction [1]. According to [2], intelligent transport systems (ITS) are advanced applications integrating telecommunications, electronics, and information technologies to provide innovative services relating to different modes of transport and traffic management. However, it should be remembered that the basic elements that allow to obtain information for this type of system are all kinds of sensors. In order to ensure the development of ITS, one should not forget about educating engineers, who create such systems, in the field of knowledge about sensors.

The education of future engineers in the field of operation and use of sensors is largely based on hands-on laboratories. They provide live interaction with the sensors and instruments used to measure the sensor outputs and practical skills required for engineering graduates. For some students, this is an important experience as it overcomes the psychological barrier in carrying out practical work with real devices.

Due to the continuous development of various Internet technologies, nowadays it is possible to carry out experiments remotely. The approach in which the real equipment of the stand is operated and controlled remotely by means of some interface is called the remote laboratory stand (RLS). Replacing the physical system with its model leads to the creation of a virtual laboratory stand (VLS) [3]. Both approaches are widely used in engineering education for a variety of reasons, nowadays mainly because of the COVID-19 pandemic [4,5,6,7,8,9,10], but also by fully online universities [11].

A number of advantages as well as disadvantages of real, remote, and virtual laboratories have been presented in [12]. The VLS can only be implemented when a mathematical model of a phenomenon or an object is available, which is its great limitation. On the other hand, if a mathematical model is available, depending on its complexity, the stand may present general principles, e.g., of using instruments (such as a caliper or micrometer) in dimensional and geometrical metrology [13] or be very similar to the real stand by using virtual reality [14]. Therefore, VLSs can be a good preparation tool for the proper laboratory exercise.

While the VLS consists mainly of software (not taking into account the computer it runs on), the RLS is a hybrid system that utilizes hardware and software. As a result, the cost of implementing an RLS is higher. In addition, the implementation of an RLS becomes troublesome when its operation requires mechanical movements. However, the implementation of a laboratory stand such as an RLS allows for sharing resources by different universities at a later time [15,16,17], as well as using them in distance learning for students with disabilities.

This article presents the general concept and practical implementation of laboratory stands developed at the Institute of Metrology, Electronics and Computer Science of the University of Zielona Góra (UZ), as part of the ReLabEMA project [18], in the case of testing sensors for electrical and non-electrical quantities. The presented concept takes into account the subsequent use of the stands not only to carry out traditional hands-on laboratories but also for conducting them remotely. In Section 2, we present the general concept of the laboratory stand from the hardware, software, and remote access points of view, focusing on low-cost solutions. The implementation and tests of two stands are presented in Section 3. Section 4 consists of the conclusions and a comparison of the presented approach to other systems available in the literature.

## 2. Concept of the Laboratory Stand

### 2.1. Hardware

**Figure 1:**
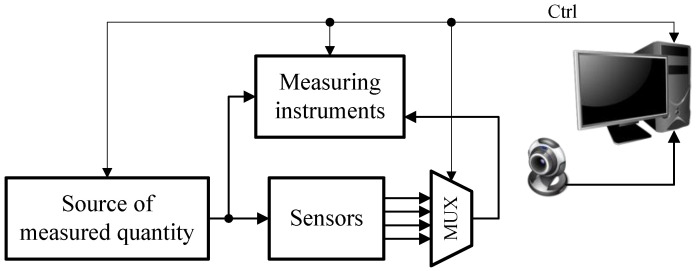
Conceptual diagram of a local and remote-operated laboratory stand.

Figure 1 shows the general concept of the hardware part of the laboratory stand for testing sensors of electrical and non-electrical quantities. The testing of sensors primarily consists of determining their transfer function, in which the tested quantity is a certain parameter of the output signal from the sensor and the forced quantity is a specific parameter of the input signal of the sensor. If sensors of electrical quantities are tested, then the stand should be equipped with an appropriate source of such a signal in the form of a voltage generator or a voltage or current calibrator. In the case of systems for testing sensors of non-electrical quantities, the sources should be calibrators of appropriate non-electrical quantities, e.g., a calibration furnace for temperature sensors, a pressure calibrator for pressure sensors, a climatic chamber for humidity sensors, or some mechanical systems for displacement and force sensors. The input signal of the sensor may or may not be measured. If the generating device produces the signal with an appropriate accuracy, the set value is taken as the value of that quantity. Due to the remote operation of the stand, each of such devices must be able to be controlled by a computer.

Regardless of whether it is an electrical or non-electrical quantity sensor, its output signal is predominantly electrical, so a suitable measuring instrument is required to measure it. In the case of sensors for electrical quantities, this measuring instrument can also measure the input quantity of the sensor if necessary. Due to the remote operation of the stand, the instrument used should also have an interface enabling its control by a computer.

To show the differences between sensors of the same quantity but of different types, it was assumed that more than one sensor could be tested on a given stand. In such a case, it is necessary to set up a measurement path by means of multiplexers. In the case of the remote operation of the stand, it can be performed with the use of relays placed on a properly designed printed circuit board, to which measuring devices, sensors, and generators of the measured quantity are attached. Controlling the relays requires a certain group of digital control signals.

Therefore, the implementation of a laboratory stand that will be operated remotely requires measuring instruments with interfaces as well as digital and analog control signals. These requirements are met in various ways. PXI systems [19,20], which are characterized by high flexibility in configuration but also at a significant price, are relatively often chosen. Sometimes data acquisition systems are used [21,22], but they are also expensive. In order to reduce costs, some solutions use Raspberry Pi microcomputers [23,24,25] or microcontrollers [26]. In the presented concept of the stand, the Analog Discovery 2 (AD2) module was used both as the measuring instrument and the control system. The module has two analog inputs that can measure voltage signals in the range of ±25 V. There are also two analog outputs that enable the generation of signals in the range of ±5 V. Both analog inputs and outputs have a resolution of 14 bits and a sample rate of 100 MS/s. There are 16 digital lines that can be used for both control and digital signal acquisition.

The AD2 module is connected to the computer via the USB interface, and its functionality is largely realized by the software running on the computer. The software provided by the manufacturer implements a very wide range of functionalities, making it possible to test various options required in a created laboratory stand without the need to create software on one’s own. On the other hand, the manufacturer of the module provides libraries for various programming languages that allow the user to create their own software with a graphical user interface dedicated to specific tasks. This feature and the price of the AD2 determined its use as a measurement and control device.

Because, according to the assumption, the stand should enable local and remote work, the hardware part also includes a camera connected to the computer, whose task is to present the view of the station. This is especially important when the stand includes mechanical elements. Additionally, if the stand is equipped with measuring instruments with displays, it is possible to observe the results presented on the displays. Therefore, despite remote work, the student is not deprived of real contact with the instruments.

### 2.2. Software

One of the most frequently used environments for creating software that manages the work of remote laboratory stands is the LabVIEW environment [3,27]. Most likely, this is due to the fact that such stands are actually measurement and control systems, and the LabVIEW environment is dedicated to developing software for this type of system. Another reason for this may be the fact that this environment uses a graphical programming language, where the programming is performed by wiring together graphical icons on a diagram, defining a specific data flow and its processing. Because, in many cases, different processes and tasks are often modeled and visualized as a data flow, it is easier in the LabVIEW to translate different concepts into software.

In the presented concept, the laboratory stand management software can be created in any programming environment and with the use of any programming language. The basic condition is that the programming environment used should allow the easiest possible implementation of the operation of devices included in the stand.

### 2.3. Remote Access

Some implementations of remote laboratory stands are based on learning management systems (LMS), portals, or other educational environments [28,29]. This approach requires a significant amount of work and makes sense when the number of stations is large and when they can be operated remotely without supervision in a laboratory room. In the case of laboratory stands with sensors, this is not possible. If the stand concerns current sensors, their work is tested, for example, with the use of a calibrator generating currents of up to 5 A. In the case of displacement sensors, the stand consists of mechanical systems which, in the event of a failure, may damage the tested sensors. In these cases, for safety reasons, supervision of the stands is necessary.

A much simpler solution is to provide remote access to the computer that manages the hardware part of the stand. From the Windows OS point of view, this can be performed via the Microsoft Remote Desktop. However, this approach has one major drawback: a public IP address must be assigned to the computer managing the stand. From the point of view of universities, for security reasons, services that manage IT infrastructure often limit the number of such computers, which makes it difficult to implement remote access in this way.

Another way to ensure the possibility of a connection with a computer in the network is to run a virtual private network (VPN) between a computer shared in the network and a computer intended for remote work [30]. In this case, the computer sharing the resources does not need to be connected to the network via a public IP address. An encrypted and secure link is established for the connection, which allows network traffic between computers. A user working at a remote computer accesses the shared computer as if they were working on the same network. However, this solution has some disadvantages from a security point of view, because once such a connection is established, the remote user gains access to the network of the computer sharing the resources. It is difficult to control this connection, and for this reason, network administrators usually do not allow it to run unauthorized.

Remote access to the laboratory stand without assigning a public IP address to the computer managing its work is also possible using remote desktop programs. There are several solutions, such as TeamViewer or AnyDesk. This type of solution requires running special software on the computer that provides the desktop, which allows to remotely connect to the desktop of that computer. On the side of the computer intended for remote work, a special software that allows to connect to the computer sharing the resources should also be run. After the connection is established, the user of the remote computer gets access to the desktop of the computer sharing the resources. However, the use of these programs requires the payment of license fees. There are also solutions that perform remote desktop functions in the form of an add-on for a web browser. This solution can be run in the Chrome web browser. The Chrome browser and the add-on that performs remote desktop functions—the Chrome Remote Desktop—must be installed on the sharing and remote computer. The add-on allows to work with the desktop of a remote computer after activating this option on the computer sharing the screen. The described solution does not require the payment of license fees but requires a Google account.

## 3. Implementation of the Laboratory Stands

Based on the presented concept, two laboratory stands were made. One of them is designed for testing current sensors, and the other is for testing displacement sensors.

### 3.1. Hardware

**Figure 2:**
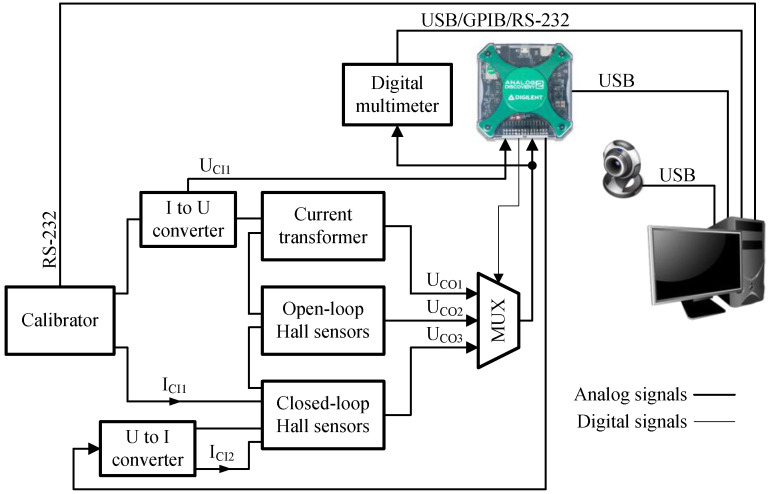
Components of a laboratory stand for testing current sensors.

**Figure 3:**
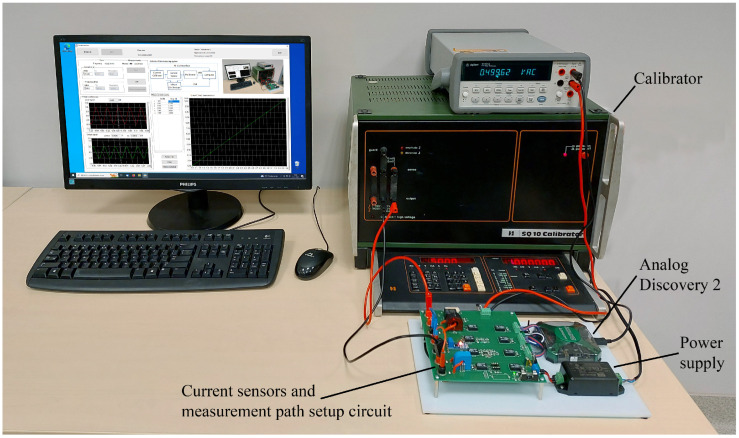
Real view of a laboratory stand for testing current sensors.

At the current sensor testing stand (CSTS), three types of sensors are tested: current transformer, open-loop Hall-effect sensor, and closed-loop Hall-effect sensor. Their part numbers and parameters are presented in Table 1. Figure 2 shows the elements and their interconnection of the CSTS. In turn, Figure 3 shows the real view of the stand. The source of the input signal for the tested sensors is the universal SQ10 calibrator, which enables the generation of currents up to 10 A, in the band up to 5 kHz, with a basic error of ±0.05%. Its setting determines the correct value of this quantity. The output signals from the sensors are measured by the AD2. If greater measurement accuracy is needed, it is possible to measure these signals with a multimeter. The selection of the signal to be measured is made by means of multiplexers based on relays. The relays are controlled from the AD2 digital outputs. As the input signal of these sensors is current, this is also additionally measured with AD2 (the current is indirectly measured as a voltage drop across a resistor). This stand also includes a voltage-controlled current source, which allows the LTS 6-NP sensor to be tested as a current difference sensor.

At the displacement sensor testing stand (DSTS), four types of sensors can be tested: two linear variable displacement transducers (LVDTs) and two proximity sensors. Their part numbers and parameters are presented in Table 2. The LVDTs differ in the type of supply and output signal. One of them is supplied with a DC voltage, and also the output signal from the sensor, proportional to the displacement of its core, is a DC voltage. The second one is supplied with an AC voltage and the output signal is also an AC voltage, the amplitude of which is proportional to the displacement of the sensor core.

**Figure 4:**
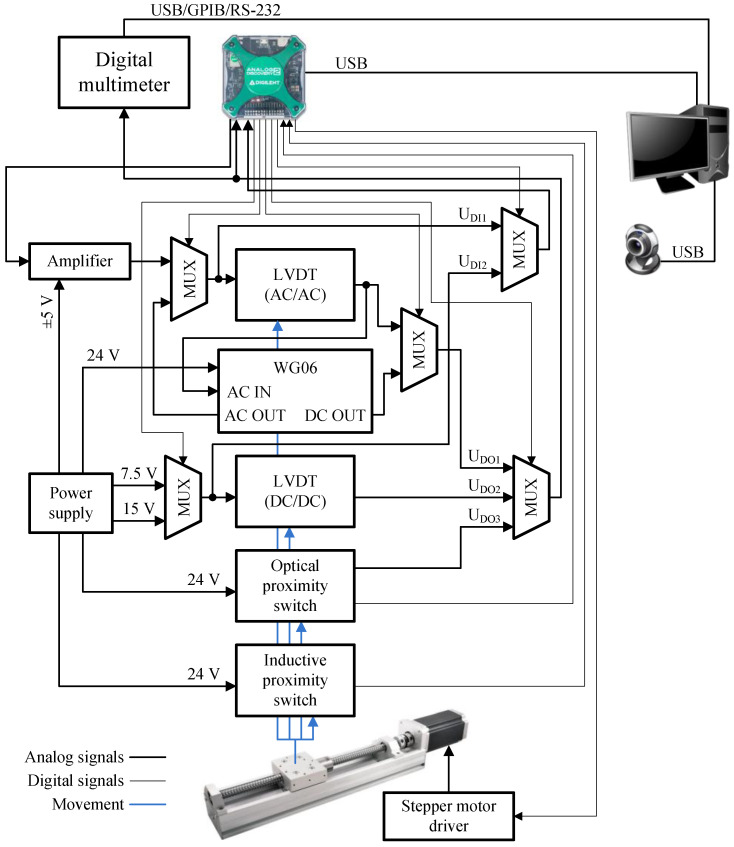
Components of a laboratory stand for testing displacement sensors.

**Figure 5:**
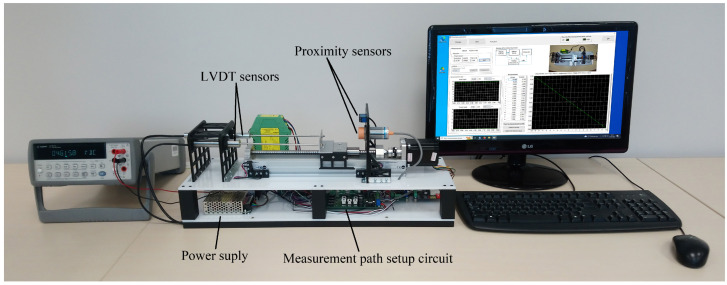
Real view of a laboratory stand for testing displacement sensors.

Figure 4 shows the elements and their interconnection of the CSTS. Its real view is shown in Figure 5. LVDT DC/DC sensor can be tested at two different supply voltages (5 V and 7.5 V). In turn, the LVDT AC/AC sensor can be tested while working with a dedicated WG06 conditioner, which enables the sensor to be powered with an AC voltage of 2 V RMS and a frequency of 5 kHz, simultaneously converting the AC output voltage to DC voltage in the range of ±10 V, depending on the location of the sensor core. The LVDT AC/AC sensor can also be powered by an amplifier that generates a voltage signal with a frequency ranging from 100 Hz to 6 kHz. This enables to carry out a test to determine the frequency of the supply voltage at which the tested sensor is characterized by the highest sensitivity.

Same as in the CSTS, the output signals from the sensors tested on the DSTS are measured by the AD2 or multimeter. Moreover, the selection of the signal to be measured and the supply source is made by means of multiplexers based on relays. The linear guide with a stepper motor is designed to move the cores of the LVDTs, the light reflecting surface for the optical sensor, and the metal surface detected by the inductive proximity sensor. The design of the linear guide and the stepper motor driver provide a resolution of linear shifts equal to 0.025 mm. At the ends of the linear guide, limit switches are placed to prevent the carriage from hitting the sensors in the case of its uncontrolled operation.

### 3.2. Software

The hardware part in both laboratory stands is managed by computer programs with similar functionality and graphical user interface, created in the LabWindows/CVI environment. Each of the programs was written in two language versions (Polish and English) selected immediately after starting the program and has a number of tabs that can be switched sequentially, which forces the proper procedure for operating the stand. The individual tabs are responsible for the following functions:

- System configuration—selection of the sensor for testing, its application system, and measuring instrument;
- System calibration—checking the correct operation of the current source (CSTS) or positioning the linear guide (DSTS);
- Measurements—selection of the test type and measurement mode (manual, automatic).

**Figure 6:**
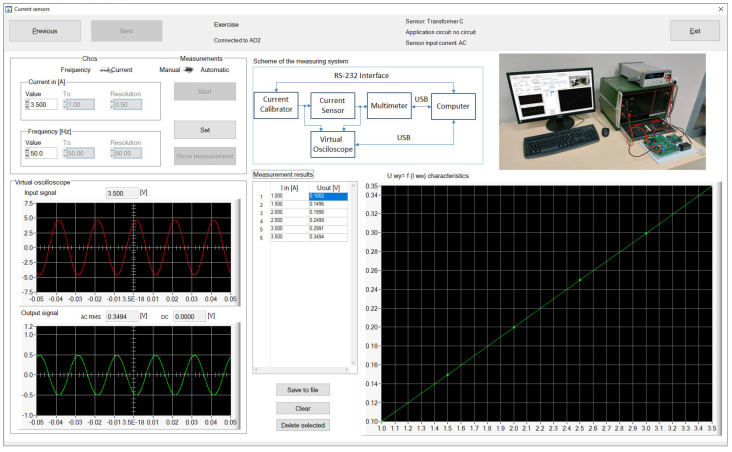
Screenshot of the CSTS control program window.

**Figure 7:**
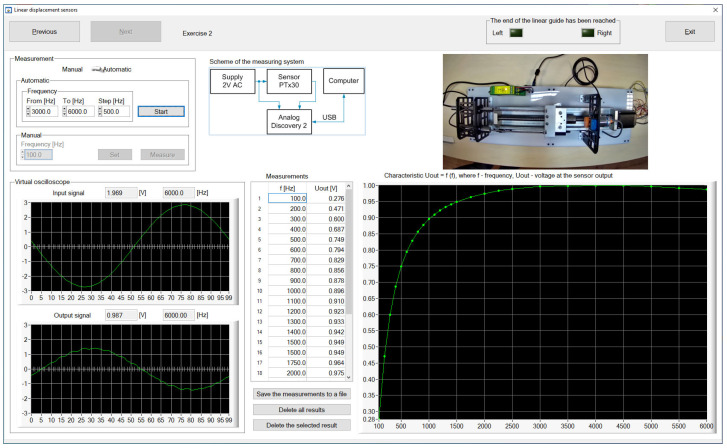
Screenshot of the DSTS control program window.

The measurement results are presented both in the form of tables and graphs. They can also be saved to a CSV file for further processing and reporting. Figure 6 shows the screenshot of the CSTS control program while determining the transfer function of the TS100 sensor for the selected frequency of the current. The program window contains the settings of the input parameters of the sensor, input and output current waveforms, and a summary of the measurement results in a tabular and graphical form. Figure 7 shows the screenshot of the DSTS control program while determining the characteristic curve of changes in the output voltage signal of the PTx30 sensor as a function of the frequency of the voltage signal supplying the sensor.

In the case of both stations, the measurement system diagram is also presented in the window of programs managing their work, and remote work is supported by the view from the camera directed at the stand.

### 3.3. Remote Access

Remote access to the laboratory stand is not implemented directly by the program controlling the stand, but it was decided to use the technique of remote access to the computer by means of the so-called remote desktop, where the user from their computer can see and control the remote computer screen. Because the UZ is a subscriber of the Google Workspace for Education and for almost 2 years the Google Classroom has been widely used at the UZ by teachers and students as a kind of an LMS, it was kind of natural to use the tools available in this subscription to create remote access.

In the presented solution, direct audio and video contact with the student is provided by the Google Meet application launched in any web browser. By creating meetings in the Google calendar, the teacher determines the time range that the student will be assigned to work on the laboratory stand. In order to participate in the meeting, the student must log in to their Google account assigned to them by the UZ. Because the teacher determines the students who will participate in the meeting, user authentication and access are verified at this level.

Remote access to the computer on which the laboratory stand control program runs is provided by the Chrome Remote Desktop, which is an extension to a web browser. This extension works fine with the most popular browsers and is fully cross-platform, providing remote secure access for Windows, Mac, and Linux users.

Before the access to the computer is made available, it must be logged into the Google account also in the browser running on the computer in the laboratory. Therefore, it is best if such a computer has a separate Google account that can be used by all teachers.

The Chrome Remote Desktop enables to access a computer remotely or share the computer with someone else. In the first case, users need to log in on the local computer and the remote computer with the same Google account. Because the computer in the lab is logged into the account assigned to the laboratory stand, and the student is logged into their account on the remote computer, this approach cannot be used. In the second case, the computer may be shared with a user logged in to a different Google account. It is enough for the user (in this case the teacher) to generate the access code and send it to the student via an e-mail or the Google Meet chat. After entering the code, the student waits for the access to be accepted by the teacher. When this is performed, remote access continues for 30 min. This time interval cannot be changed by the user. After this time, the access is blocked and the teacher has the option to extend it by another 30 min interval. The teacher can also terminate access to the computer in the laboratory at any time.

### 3.4. Tests

The Rapid Application Development (RAD) methodology [31] was used in the process of the software development that controls the hardware parts of the laboratory stands. RAD is a multiphase adaptive software development model based on prototyping and quick feedback, with less emphasis on specific planning. In the first phase, general requirements for the software were defined, such as the appearance of the user interface and action scenarios during its operation. In the next phase, a prototype of the software application was developed and made available for testing. Information on the operation and use of the application collected from users testing the application allowed for the implementation of corrections and improvements.

One of the stages of the ReLabEMA project was a pilot workshop, during which students carried out tests of the laboratory stands. The venue for the workshops was Tallinn University of Technology (Estonia). The workshop was attended by 40 students from the universities listed in Table 3. Each of the laboratory stands was tested by 4 groups of students, which included people from different countries and universities. Each test lasted about 2 h and consisted of carrying out a laboratory exercise according to the provided manual. In the laboratory, the exercises were supervised by two teachers (one teacher at each of the two stands). They assessed the fluency of using the application by different groups of students, which allowed to draw conclusions regarding the understanding of the exercise. The comments of these teachers were taken into account when modifying the exercise manuals. The third teacher was with the group of students performing the exercise remotely, taking notes and providing technical assistance.

After completing the exercise, the teacher conducted an interview with the students performing the exercise, asking them a series of questions. The asked questions concerned the following topics:

- The quality of the remote connection;
- The problems with the software application caused by remote work;
- The ergonomics of the user interface of the prepared software;
- The noticed bugs in the operation of the software;
- The comprehensibility of the user interface;
- The clarity of the presented measurement results;
- The ease of recording the measurement results for their later use;
- The improvement in the application functionality.

The collected interview results allowed the following conclusions to be drawn.

- Remote work did not cause any difficulties in the operation of the software applications.
- Students did not report any serious bugs in the operation of the applications.
- The applications’ graphical interface needed to be modified due to a poorly chosen function description.
- The method of saving measurements implemented in both applications should be unified and improved.

In the tested version of the software, the video preview of the laboratory stand was carried out in a separate application. A teacher watching the students’ work noticed that this solution was inconvenient because the students, after making the settings, tried to quickly switch to the camera preview window to see the reaction of the hardware part, which was not always successful. Therefore, in the new version of the software, not only fixes related to the students’ comments were applied but also the video preview window was built into the application, which is shown in Figure 6 and Figure 7.

## 4. Conclusions

In this paper, the general concept of a laboratory stand for testing sensors of electrical and non-electrical quantities is presented. To implement the stand, only a computer with an optional USB camera and the AD2 module providing the acquisition and generation of analog and digital signals are required. In the case of remote access to the stand, active Google accounts and a computer with Internet access are required. Depending on the type of tested sensors, it becomes necessary to design and develop a certain hardware part that will allow the sensors to be connected to the AD2 and the power supply.

Based on the presented concept, two laboratory stands were prepared to perform exercises related to the testing of current and displacement sensors. These stands allow for the implementation of a full program of laboratory exercises, which so far has been carried out on manually operated stands in the Laboratory of Measuring Transducers of the Institute of Metrology, Electronics and Computer Science of the UZ. The hardware part of each workstation is operated only by a dedicated software application running on the computer in the laboratory. The teacher’s participation is limited to supervision over the work of students. The stand can be operated locally and remotely. The image from the camera helps in remote work. Remote access to the two described laboratory stands was tested by 40 students, which made it possible to make sure that the adopted concept of remote access to the stand works in practice and to identify various shortcomings of the software controlling the hardware of the stand. Despite the significant distance between the users and the laboratory, there were no distortions in the operation of the stands nor the video transmission.

Regarding the solutions of the remote laboratory systems presented in [19,20,21,22,23,24,25], the developed systems are characterized by a universal, simple, and cheap hardware structure. The systems do not require specialized communication interfaces or expensive laboratory equipment. The multimeter, proposed in both systems, is an optional instrument. The AD2 module used for the control and measurement communicates with the computer via a generally available USB interface. As in the case of other systems of remote laboratories using real test objects and real instruments, there is also a need in the presented concept to properly prepare the test object together with the systems of the controlled switches that allow changing the system configuration or setting values of the quantities used in the systems.

Access to laboratory stands using the Chrome Remote Desktop has one significant advantage over access via a website. The teacher observes the computer screen with the application operated by the student (mouse movements, entered parameters, and measurement results). Therefore, the student can be provided with hints from the teacher in real time (using voice communication or chat) and even correct the settings, as the teacher has direct access, in relation to the remote user, to the desktop of the computer running the program controlling the stand. Performing exercises by students present locally in the laboratory does not require changing the software that manages the stand. The Chrome Remote Desktop will not be used in this case.

The presented concept of the hardware part of the stand can be used to build other teaching or research stands in the areas of automation, electronics, electrical engineering, mechatronics, and metrology.

## Figures and Tables

**Table 1:** Parameters of the tested current sensors.

Sensor		Input	Output	Basic
Type	Part No.	Current (A)	Signal	Error (%)
Current transformer	TA 100	5	5 mA	±1
Open-loop Hall-effect sensor	ACS 712	5	2.5 ± 0.925 V	±1.5
Closed-loop Hall-effect sensor	LTS 6-NP	6	2.5 ± 0.625 V	±0.7

**Table 2:** Parameters of the tested displacement sensors.

Sensor		Range	Output	Digital	Linearity
Type	Part No.	(mm)	Signal (V)	Output	(%)
LVDT (AC/AC)	PTx30	±30	0–1 RMS	–	≤0.5
LVDT (DC/DC)	PIz20	±20	±5	–	≤0.5
Optical proximity switch	FT20RA-60-F-K4	20–80	10–0	PNP/NPN	–
Inductive proximity switch	LJ30A3-15-Z-CY	15	–	PNP/NPN	–

**Table 3:** Pilot workshop participants conducting tests.

University Name	Country	No. of Students
Politehnica University of Timisoara	Romania	4
Silesian University of Technology	Poland	8
Tallinn University of Technology	Estonia	5
University of Applied Sciences Mittelhessen	Germany	4
University of Zielona Góra	Poland	5
Vilnius Gediminas Technical University	Lithuania	6
Zespół Szkół Technicznych in Wodzisław Śl.	Poland	8

## Data Availability

Not applicable.
